# Improving Adolescent Health Risk Assessment: A Multi-method Pilot Study

**DOI:** 10.1007/s10995-016-2070-5

**Published:** 2016-07-12

**Authors:** Lindsay A. Thompson, Martin Wegman, Keith Muller, Katie Z. Eddleton, Michael Muszynski, Mobeen Rathore, Jessica De Leon, Elizabeth A. Shenkman, the Health IMPACTS for Florida Network, Akilah Pope, Ana Alvarez, Anabella Torres, Ann M. Usitalo, Anneka Johnson, Anya Monroe, Ayesha Mirza, Brenda L. Figueroa, Brittany Brown, Carol Fulton, Carolyn Campbell, D. Paul Robinson, Debra S. Andree, Donald Michel, Tamara-Kay Tibby, Temple Robinson, Elizabeth Hengstebeck, Elizma Mercier, Ellie Lawson, Ernestine M. Lee, Felechia Gant, Heather Morosco, Heidi Vetter, Jacquelyn Nystrom, Jennifer Manuel, Jennifer Tickal Keehbauch, Joseph F. Savona, Joseph T. Sherrel, Judith R. Banks, Judy Yelvington, Keith Conger, Kettline Esperance, Kimberly Meurer, Le’Chantel Bright, Lionel A. Henry, Lisa Tumarkin, Marianna Towler, Mark B. DiDea, Melissa Flora, Miraqa Nizar, Natasha King, Nichelle Richards, Nicholas D. Peterkin, Nickeey K. Malcolm, Nicole L. Cameron, Nizar F. Maraqa, Olga “Pat” Canete, Olin B. Mauldin, Pauline Rolle, Penelope Tokarski-Savona, Rosimar Bernabe, Saniyya Mahmoudi, Silang Defour, Susan Moore, Tajvar Goudarzi, Tawanda Washington, Taylor Mangrum, Tiffany Welch, Tongela Davis, Tracy Ingram, Mahmoud Enani, Maryum Khan, Michelle Vinson, TaJuana Chisholm

**Affiliations:** 1 Department of Pediatrics, College of Medicine, University of Florida, 1699 SW 16th Ave, Gainesville, FL 32608 USA; 2 Department of Health Outcomes and Policy, University of Florida, Gainesville, FL USA; 3 College of Medicine, Florida State University, Tallahassee, FL USA; 4 Department of Pediatrics, University of Florida, Jacksonville, FL USA

**Keywords:** Adolescent health services, Preventive health services, Health information technology, Counseling/standards, Health care surveys, Health behavior, Quality improvement, Practice-based research network, Patient-reported outcomes

## Abstract

*Objectives* Given poor compliance by providers with adolescent health risk assessment (HRA) in primary care, we describe the development and feasibility of using a health information technology (HIT)-enhanced HRA to improve the frequency of HRAs in diverse clinical settings, asking adolescents’ recall of quality of care as a primary outcome. *Methods* We conducted focus groups and surveys with key stakeholders (Phase I) , including adolescents, clinic staff and providers to design and implement an intervention in a practice-based research network delivering private, comprehensive HRAs via tablet (Phase II). Providers and adolescents received geo-coded community resources according to individualized risks. Following the point-of-care implementation , we collected patient-reported outcomes using post-visit quality surveys (Phase III). Patient-reported outcomes from intervention and comparison clinics were analyzed using a mixed-model, fitted separately for each survey domain. *Results* Stakeholders agreed upon an HIT-enhanced HRA (Phase I). Twenty-two academic and community practices in north-central Florida then recruited 609 diverse adolescents (14–18 years) during primary care visits over 6 months; (mean patients enrolled = 28; median = 20; range 1–116; Phase II). Adolescents receiving the intervention later reported higher receipt of confidential/private care and counseling related to emotions and relationships (adjusted scores 0.42 vs 0.08 out of 1.0, *p* < .01; 0.85 vs 0.57, *p* < .001, respectively, Phase III) than those receiving usual care. Both are important quality indicators for adolescent well-child visits. *Conclusions* Stakeholder input was critical to the acceptability of the HIT-enhanced HRA. Patient recruitment data indicate that the intervention was feasible in a variety of clinical settings and the pilot evaluation data indicate that the intervention may improve adolescents’ perceptions of high quality care.

## Significance

To address the known gap in adolescent risk assessments in primary care visits, this pilot study developed an intervention that systematically implemented a technologically-enhanced health risk assessment for adolescents. The combination of stakeholder engagement with adolescents and all kinds of providers, combined with the technology-based support systems provided a structure for the implementation of systematic screening. In follow-up, adolescents who received the intervention felt their care was more private and confidential than the ones who received usual care.

## Introduction

Adolescence is an important period during which risky behaviors (i.e. alcohol, drug and tobacco use, sexual activity), and mental health concerns often develop, contributing to adolescent morbidity and mortality and increasing the risk of developing lifelong chronic conditions [5]. Provider-initiated health risk assessment (HRA) screening and counseling is a cornerstone of adolescent care according to the Society for Adolescent Health and Medicine, the American Medical Association, and the American Academy of Pediatrics [9, 10, 30]. Unfortunately, numerous studies have documented low levels (3–25 %) of adolescent HRA in primary care [3, 15, 27]. Inconsistent assessment is often due to time constraints, confidentiality concerns, and a lack of office systems to facilitate completion [2, 15].

While studies have detailed how to improve systemic implementation of adolescent HRA and counseling [25], a new emphasis on health information technology (HIT) may additionally ensure systematic integration of screening during clinic visits and increase evidence-based practice. HIT has been shown to increase provider self-efficacy and confidence in conducting HRAs, enhancing counseling and referrals [12, 24, 29, 33], and improving teens’ recall of their visits [23]. Additionally, incorporating stakeholder perspectives into intervention designs can increase the ecological validity, uptake and sustainability of an intervention [11, 17] and is supported by current efforts of patient-centeredness. The two primary implementation goals of our study were to: (1) engage clinicians, staff, and adolescents from outpatient clinics in the design of an HIT-enhanced HRA (Phase I); and (2) pilot test the HIT-enhanced HRA in a broad and diverse practice-based research network to establish its feasibility (Phase II). As a primary outcomes goal, we evaluated adolescents’ recall of their primary care visits using an established patient quality of care survey (Phase III) to obtain a patient-centered perspective on the intervention.

## Methods

### Methods Overview

This pilot study was conducted within Health IMPACTS (Integrating Medical Practice and Community-based Translational Science) for Florida, a practice-based research network (PBRN) managed jointly by the University of Florida and Florida State University. Institutional Review Boards at both institutions, four hospitals and the Florida Department of Health approved this study. We conducted a multi-phase, multi-method feasibility study to design an HIT-enhanced HRA intervention (Phase I), implement the intervention within the PBRN (Phase II), and obtain patient-centered outcomes information about the acceptability of the intervention (Phase III). Feasibility was defined as the ability of practices to implement the protocol and enroll patients in the protocol, which included use of the HIT-enhanced HRA. A subset of the adolescents also participated in the Phase III pilot evaluation, which collected patient-reported outcomes via a telephone survey following the adolescent’s clinic visit to assess their experiences with care.

### Phase I Methods: Gathering Stakeholder Perspectives

#### Adolescent, Provider and Staff Recruitment

During Phase I, focus groups and surveys were conducted with adolescents, clinic staff, and providers as key stakeholders. Specific information was sought from each activity and group; data were integrated into the development of the customized HIT tool and in the design of the overall implementation strategy. Recruitment of adolescent focus group participants using Florida’s Medicaid and State Children’s Health Insurance Program databases resulted in the recruitment of 35 racially/ethnically diverse adolescents to 8 focus groups and is reported in its entirety elsewhere. [13, 16, 37] Office staff and providers were recruited to participate in semi-structured focus group interviews led by trained moderators and co-moderators [1]. The purpose of these interviews was to acquire information about offices’ and practitioners’ past experiences and attitudes about comprehensive HRA screening and counseling and elucidate barriers and facilitators. Open-ended questions were intentionally used to encourage discussion and elicit new ideas. Sixty-five participants (pediatric and family medicine physicians, residents, nurse practitioners, nurses, medical assistants, nursing assistants, front office staff and business managers) participated in nine focus groups. All discussions were recorded, transcribed and analyzed to identify major themes, using ATLAS.ti, a qualitative data management software (Version 7.0, Berlin, Germany 2011). These focus groups informed the design of the HIT tool and overall implementation strategy.

#### Practice and Physician Surveys

The study team recruited a convenience sample of pediatric and family medicine practices in Gainesville (n = 4), Jacksonville (n = 7), Orlando (n = 6) and Tallahassee (n = 5), Florida. The practices were functionally diverse and included academic and non-academic clinics as well as Federally-qualified health centers, representing patients with commercial and Medicaid insurance. Practices chose who would attend the focus groups depending on who would be implementing the HIT-enhanced HRA in their clinic, so attendee type varied from clinic to clinic. Demographic information on these attendees was not recorded. Practice-based surveys gathered relevant practice-level information from medical directors and office managers (N = 22; response rate = 95.7 %). Providers at these practices answered survey questions on current practices related to HRA screening and counseling (N = 80; response rate = 73.4 %; see Table 2). Domains included behavioral health risk screening, counseling, and referral practices, as well as knowledge of related community resources. Additional items captured practitioner demographics (e.g. age, gender, race/ethnicity, years in practice) and practice characteristics (e.g. specialty, population demographic served, staffing, electronic records and communication, quality improvement orientation, accessibility). All surveys are available upon request.

### Phase II Methods: Design and Implementation of HIT-Enhanced HRA

#### Intervention Design

Drawing from Phase I with input from key stakeholders, this phase consisted of the integration of stakeholder perspectives, recruitment of practices and adolescents to establish if the pilot was feasible. Specifically, we adapted a commonly used HRA, the Guidelines for Adolescent Preventive Services (GAPS) [9] to create an 82-question (75 clinical and 8 demographic) HIT-enhanced HRA accessed via tablet (iPads). We chose this traditionally paper-based screener because it is the most widely recognized in clinical practice; represents a framework for the delivery of comprehensive care; is supported by organizations most strongly associated with adolescent care such as the American Academy of Pediatrics and the Society for Adolescent Health and Medicine; and is complementary to the mnemonic, HEADSSSS (Home, Education and employment, eating, Activities with peers, Drugs, Sexuality, Suicide and depression, Safety, Spirituality and Strengths) when performing face-to-face screening. We made further modifications to include aspects of the patient experience that did not exist when the original GAPS was written (e.g. cyberbullying). The tool is available upon request. The literacy level of the HIT-enhanced HRA was 5.8 according to the Flesch-Kincaid Grade level and was only available in English. The adolescents in the pilot test of phase 1 took no longer than 15 min to complete it. While the focus groups were demographically, racially and ethnically diverse [18, 22, 37], only those who could read English were enrolled in this study. The HIT-enhanced HRA was embedded in novel software with restricted access to ensure security and confidentiality. The software aggregated the responses into a real-time report separately available via secure Internet connection, highlighting high-risk behaviors. Reports could be printed or uploaded into the adolescent’s medical record. Furthermore, the HIT-enhanced HRA generated a list of geo-coded links to relevant community resources that could be printed, scanned into a smartphone via quick response (QR) code, or e-mailed to the adolescent. These geo-coded resources were selected by zip code to minimize travel-related barriers that adolescents face, were free or low cost, and were available for every risk screened in the HRA.

The overall premise of the study was to use the HIT platform across diverse sites. Due to the variety of clinical practices participating in this study, there were eight different EHR systems represented and 35 % of the clinical practices used a paper-based medical record system. For this reason, the web-based application was developed to function outside any EHR. The web-based system was primarily accessed through Wi-Fi-enabled iPads and iPads with cellular data service were made available to clinics without Wi-Fi. Although this web-based application required the providers to log into a separate system to review the HRA, all practices were able to participate in the study equally and the basic methodology could be incorporated into any clinic’s workflow. Additionally, the HRA questions and responses could be exported to a PDF to allow the survey to be integrated into an electronic or paper-based health record. This also positively impacted the scalability of the intervention, as it could be conducted in almost any health care delivery setting regardless of medical record system or IT infrastructure. Practices varied in their implementation of the study: some practices had all providers involved, others only one of many providers, and others still had nursing champions that made recruitment productive when present but slowed when absent.

The cost of the project was multifaceted. The University of Florida made a significant investment, along with State of Florida and National Institute of Health funds, to create the web-based application, which was designed to support any point-of-care screening study. This was the largest cost by far and only a one-time cost. Additional practice-specific costs included printing study materials and the need for staff time to help embed the intervention into the clinic’s workflow and support the practice through periodic practice site visits. This model scales well, as additional practices did not significantly increase the overall cost of the project. More clinical practices implementing the intervention and using the software actually decreases the cost per practice and increases the cost effectiveness of the intervention. Future practices that might want to use this platform would only have to cover costs for tablets and practice facilitation.

#### Implementation: Adolescent Recruitment

A convenience sample of adolescents 14–18 years old and their parents were approached during primary care visits, which were typically well-child visits. Study coordinators worked as practice facilitators, training clinic staff on the protocol and making frequent visits to each clinic to ensure fidelity and to address any implementation issues. Fidelity monitoring was systematically reviewed weekly and issues were resolved in a variety of ways. Given practice differences, study coordinators had to work through site-specific adaptations, figuring out which were acceptable and which were too significantly different from the study protocol to be allowed. The most provocative example was the need to physically separate the parent and adolescent to allow privacy in the adolescents’ completion of the HRA. One clinic, for example, had their waiting and exam rooms on different floors and the staff was uncomfortable separating the adolescent from the parent. That clinic could only participate if they provided the adolescent with privacy in which to complete the HRA, allowing flexibility in how the practice complied, but not whether they complied with the request for private space. This provided a template for other clinics’ possible adaptation of separating the parent and adolescent.

Adolescents were presented with an informed consent document via tablet for either parental consent and teen assent or teen consent depending on the age of the adolescent. Three participating hospital-affiliated clinics also required paper consent forms. Two clinics in the most geographically diverse region (Jacksonville) were asked to be comparison sites while twenty clinics participated in full, and all adolescents and parents were approached similarly. Adolescents at comparison sites answered only seven demographic questions prior to their primary care visit related to age, gender, and race/ethnicity. They did not receive the HIT-enhanced HRA via tablet but may have participated in an HRA if that office/provider usually administered one although this was reportedly low overall from all clinics (see Results, provider and practice surveys, below).

### Phase III Methods: Patient Reported Outcomes

#### Patient Reported Outcomes: Adolescent Recruitment

A primary outcome goal was to obtain adolescent perspectives about their experiences during their primary care visit related to the HRA, counseling and privacy. Four to six weeks post-visit, adolescents recruited from nine intervention sites and two comparison sites were administered the Young Adult Health Care Survey (YAHCS) [3, 18] by phone. These nine intervention sites were selected due to their full implementation by June 1, 2012. The YAHCS measures the quality of adolescent preventive care and has strong construct validity and reliability [3]. Adolescents who completed the YAHCS received a $15 gift card to a local merchant.

#### Patient Reported Outcomes: Analysis

The YAHCS survey contains eight quality domains that ask adolescents about how their provider performed screening and counseling about: (1) prevention of risky behaviors; (2) sexual health; (3) weight, diet and exercise; (4) behavioral health; (5) provision of private and confidential care; (6) helpfulness of the counseling; (7) communication and experience of care; and (8) overall experience [3]. Even though screening and counseling are different aspects of care, they are often integrated during clinical encounters, a characteristic reflected in measurement tools assessing quality of care (e.g. the YAHCS). Thus while the HIT-enhanced HRA provided additional screening in an asynchronous fashion, this study utilized the meta-concept of integrated screening and counseling. Future work might seek to disentangle these related constructs, for example assessing if the reduction in need for provider-conducted screening is accompanied by a greater focus on counseling. For display in Table 3 and comparability, we scaled the domains to fall between 0 and 1 (with scores closest to 1 representing higher levels of screening performed). Please see “Appendix” for clarification.

We report the adjusted domain scores using a mixed-effects model to account for correlation within clinics and unequal numbers of participants per clinic [22]. The fixed set of individual-level predictor variables, chosen a priori, included: gender (male/female), race-ethnicity (white, black, Hispanic and other), age in years, and intervention (yes/no). Given our goal of feasibility, we did not choose to include provider characteristics in our predictive model as this study was not powered to detect provider effects within clinics since it would be difficult to ascribe a clinic-wide change to one care provider. All analyses were performed using SAS (version 9.3, SAS Institute Inc., Cary, NC). Compound symmetry was assumed in the analytic methods, meaning that participants are exchangeable within clinic, such that any two participants’ responses are equally correlated. Tests of fixed effects were used with a level of α < 0.01 to assess significance in order to account for separately evaluating responses in the 8 domains of the YAHCS screener. Least squares means of each modeled domain score were reported for these selected predictors (referents include male, white and mean age at 16.9 years). R-squared values were computed for the full models and for the intervention fixed effects. An analysis of residual covariance was performed for each YAHCS sub-domain to test how consistently clinics performed. We calculated intra-class correlation coefficients and confidence intervals using Searle’s method [37], where a score of 0 means no variation between clinics and 1 means no clinics performed that task the same.

## Results

A summary of the results from Phase I, II, and III can be found in Table 1.

**Table 1:** Main findings by study phase

Phase I			
Adolescent focus groups (N = 35)	Provider and staff focus groups (N = 65)	Provider surveys (N = 80)	Practice demographic surveys (N = 22)
8 Focus groups	9 Focus groups	Paper/online surveys	Paper/online surveys
HRAs should be conducted in professional, clinical settings HIT-enhancement would promote honesty and confidentiality Must not replace face-to-face counseling on risk-taking behaviors (enhances conversations with providers)	*Barriers to HRAs* Time to administer, review Lack of knowledge about health resources Privacy/confidentiality *Facilitators to HRAs* Reducing time spent Long-term provider-patient relationships HIT, culturally competent tool, access to health resources	*Low levels of* Routine GAPS administration Routine depression screening *High levels of* Discussing confidentiality Asking about common risk behaviors Referrals were highest for nutrition and exercise needs and substance use, and relatively low for tobacco and alcohol use and sexual health issues	4 FQHCs, 6 private practices, 2 affiliated with private hospitals, and 10 affiliated with academic health centers 66.7 % already implemented an EHR Even distribution of adolescent patients

Phase II	Phase III (see also Tables 2 and 3)
Practices (N = 22); adolescents (N = 609)	Adolescents (N = 200) answered telephone surveys (YAHCS)
Feasibility of HIT-enhanced HRA *Adolescent patients per clinics* Mean = 28 Median = 20 Range = 1–116 Participation rate (#enrolled/#eligible ×100 %) 13.9–100 % Implementation supports feasibility of intervention	163 adolescents included in data analysis (exclusions for “other” race) 2 of 8 domains showed intervention adolescents received significantly higher counseling (1.0 represents perfect report of counseling) 1. Receipt of private and confidential care (0.42 vs 0.08 out of 1.0, *p* < .01) 2. Counseling related to depression, mental health, emotions and relationships (0.85 vs 0.57 out of 1.0, *p* < .01)

### Phase I Results: Stakeholder Perspectives

#### Adolescent Focus Groups

Results of the adolescent focus groups were recently published [16]. The adolescents (n = 34) were diverse, with half (50.0 %) male, 55.9 % African-American, 29.4 % Hispanic and evenly ranging in ages from 14 to 18 years. In summary, adolescents preferred completing HRAs in clinical, private, and professional settings, and reported that tablet technology supported their confidentiality in completing the HRA yet facilitated conversations with their providers. Adolescents admitted that they would answer less honestly in a face-to-face interview than written or tablet-based entry, even though they also strongly endorsed face-to-face discussion of health risks with their personal provider. Overall they valued trust, confidentiality, nonjudgmental care and their relationship with their practitioner.

#### Provider and Staff Focus Groups

Provider and staff focus groups revealed barriers and facilitators to HRAs. A key barrier was the time required to both administer and review HRAs and still have meaningful discussions with patients. Providers and staff agreed that using wait time for HRA completion and having software that facilitated quick review of responses with up-to-date resources would significantly reduce this barrier. Patient concerns regarding privacy and confidentiality were addressed by requiring private administration of the HRA without parents being present and through use of an electronic system that did not allow for review of answers once completed. Based on provider and office staff feedback, the use of HRAs in primary care can be expanded and improved by addressing barriers and facilitators to administration, in part through the use of HIT.

#### Provider and Practice Surveys

Provider and practice surveys offered baseline pre-intervention data on HRA use. Of the providers, 46.3 % were Family Practitioners and 47.5 % were Pediatricians; 12.5 % were African-American, 10.0 % Asian and 8.8 % were Hispanic; and the median number of years in practice was nine. Prior to implementing the HIT-enhanced HRA intervention, providers reported low levels of ‘always’ or ‘usually’ using the GAPS [9], (17.5 %; N = 14) or ‘always’ or ‘usually’ using a screening tool for depression (13.8 %; N = 11) with their adolescent patients. However, almost all providers (88.8 %; N = 71) reported ‘always’ or ‘usually’ informing their adolescent patients that anything they discuss will remain confidential unless it poses an immediate harm to themselves or others. Most providers reported that they ‘always’ or ‘usually’ asked about the following: (1) tobacco, alcohol, and other substance use, (2) sexual activity, and (3) nutrition, and physical activity. A majority of providers felt their staff was ‘very’ or ‘moderately knowledgeable’ about common adolescent health risk-taking behaviors like tobacco use and sexual activity, but most reported only “occasionally” or “rarely” referring their adolescent patients to patient education classes, support groups and/or individual counseling. Highest referral rates were reported with nutrition, depression, weight loss, and other substance use (40.0, 38.8, 37.5, 21.3 % reporting ‘always’ or ‘usually’ making referrals, respectively). Lowest referral rates were reported with tobacco, sexual activity, alcohol use, and intimate partner violence (11.3, 15.0, 16.3, 16.3 % reporting ‘always’ or ‘usually’ making referrals, respectively).

Practices were diverse, including Federally Qualified Health Centers (n = 4), private practices (n = 6), hospital-affiliated clinics (n = 2) and academic centers (n = 10). Most (66.7 %) had electronic medical records, with an even distribution of estimated numbers of 14–18 year old adolescent patients (20 % had less than 10 % of their patients as adolescents; 26.7 % had 10–24 %; 30 % cited 25–50 and 6.7 % estimated over 50 % of their patients were 14–18 years).

### Phase II Results: Feasibility of HIT-Enhanced Adolescent HRA

#### HRA: Adolescent Recruitment Results

Twenty-two academic and community practices in north and central Florida successfully recruited 609 diverse adolescents (14–18 years) during primary care visits over a 6-month period (mean patients enrolled = 28; median = 20). Data collection was completed between February 27, 2012 and March 31, 2013. Total enrollment ranged between practices from 1 to 116, with a participation rate (adolescents recruited divided by eligible adolescents who attended the clinic) ranging from 13.9 to 100 %. This range represents variation in clinic engagement with the study as well as the variation in number of adolescent patients typically seen in pediatric primary care offices.

### Phase III Results: Patient Reported Outcomes and Experiences of Care

Two hundred participants completed the YAHCS (out of 350 eligible individuals; response rate = 62.2 %) which is a response rate in keeping with similar studies of adolescents [28]. For analytic purposes and the statistical stability of the stratified models [21], respondents were excluded if they either did not report race/ethnicity or reported races or ethnicities that had less than ten members (n = 20). Additionally, a handful of adolescents were asked a shorter survey that did not contain the questions of interest for this study, (n = 17). This yielded a final dataset of 163 individuals (99 in the intervention group and 64 in the comparison group). Participants completing the YAHCS were 58.9 % female, 48.5 % White, non-Hispanic, 38.0 % Black, non-Hispanic, and 13.5 % Hispanic. Study participants were evenly distributed by age: 14 years (20.9 %), 15 years (20.9 %), 16 years (20.9 %), 17 years (19.0 %), and 18 years (18.4 %). (Also see Tables 1 and 2).

**Table 2:** Characteristics of adolescent participants and their participating primary care office, Phase 3

	Intervention group n = 99 (%)	Comparison group n = 64 (%)	Total n = 163 (%)
*Patient-level characteristics*			
Gender			
Male	45.5	34.4	41.1
Female	54.5	65.6	58.9
Age in years			
14	26.3	12.5	20.9
15	20.2	21.9	20.9
16	21.2	20.3	20.9
17	14.1	26.6	19.0
18 + years	18.2	17.2	18.4
Race/ethnicity			
White, non-Hispanic	47.5	50.0	48.5
Black, non-Hispanic	46.5	25.0	38.0
Hispanic	6.1	25.0	13.5
Self-reported Risk Behaviors			
Smoked in past 30 days	0.0	3.1	1.2
Smoked in past 12 months	4.0	4.7	4.3
Had at least one drink of alcohol in past 30 days	8.1	11.1	9.3
Had 5 + drinks in a row in the past 30 days^a^	12.5	50.0	31.2
Ever had sexual intercourse	18.2	27.0	21.6
Did not use a condom at last sexual intercourse^a^	38.9	11.8	25.7
Sad or hopeless almost every day for two weeks	14.3	20.3	16.7

Clinic-specific Information (n = 13)	Intervention group n = 11 (%)	Comparison group n = 2 (%)
What % of the patients at your clinic are enrolled in medicaid or CHIP?		
Less than 10 %	22.2	50.0
10–24 %	11.1	0.0
25–50 %	11.1	0.0
More than 50 %	55.6	50.0
What % of the patients at your clinic are between 14 and 18 years old?		
Less than 10 %	11.1	0.0
10–24 %	44.4	0.0
25–50 %	33.3	100.0
More than 50 %	11.1	0.0
*Clinic (weighted —N is the number of respondents* *=* *163)*		
What % of the patients at your clinic are enrolled in medicaid or CHIP?		
Less than 10 %	14.1	82.8
10–24 %	22.2	0.0
25–50 %	3.0	0.0
More than 50 %	60.6	17.2
What % of the patients at your clinic are between 14 and 18 years old?		
Less than 10 %	5.1	0.0
10–24 %	48.5	0.0
25–50 %	36.4	100.0
More than 50 %	10.1	0.0

^a^Calculated only for those who reported any alcohol in past 30 days or who reported ever having sexual intercourse

Table 3 displays intervention and comparison mean effects (±SE) for each YAHCS domain, scaled from 0 to 1, where increasing values indicate higher reports of preventive screening and counseling. Scores for two domains were significantly improved in the intervention group versus the comparison group. Adolescents in the intervention group reported significantly higher rates of screening and counseling for depression, mental health, emotions and relationships (mean score of 0.42 compared to 0.08, *p* < .01), as well as receiving care that was private and confidential (mean score of 0.85 compared to 0.57, *p* < .0001, Table 2). Importantly, these responses were not significantly different by gender, race/ethnicity or age. Gender differences were observed across both groups for one domain; females reported higher levels of helpfulness of screening and counseling compared to males (mean of 0.84 vs 0.61, *p* < .01).

**Table 3:** Adjusted YAHCS quality domains, scaled for comparability

	Risky behaviors		Sexual activity and STDs		Weight, diet and exercise		Emotions and relationships		Private and confidential		Counseling helpfulness		Communication and experience of Care		Health information	
Group	Mean score^a^	SE	Mean score^a^	SE	Mean score^a^	SE	Mean score^a^	SE	Mean score^a^	SE	Mean score^a^	SE	Mean score^a^	SE	Mean score^a^	SE
Intervention (adjusted)	0.36	0.06	0.43	0.07	0.65	0.05	**0**.**42**	**0**.**05**	**0**.**85**	**0**.**04**	0.69	0.05	0.52	0.01	0.64	0.06
Control (adjusted)	0.05	0.11	0.22	0.13	0.51	0.04	**0**.**08**	**0**.**09**	**0**.**57**	**0**.**03**	0.75	0.06	0.51	0.01	0.37	0.12
*p* value	0.03		0.17		0.05		<**0**.**01**		<**0**.**0001**		0.39		0.51		0.07	
Gender																
Female	0.17	0.06	0.33	0.08	0.60	0.04	0.28	0.05	0.71	0.03	**0**.**84**	**0**.**05**	0.52	0.01	0.54	0.07
Male	0.24	0.07	0.33	0.08	0.55	0.05	0.23	0.06	0.72	0.04	**0**.**61**	**0**.**05**	0.51	0.01	0.47	0.08
*p* value	0.04		0.86		0.45		0.86		0.49		<**0**.**01**		0.49		0.66	
Race/Ethnicity																
Black/African-American	0.27	0.07	0.35	0.08	0.55	0.04	0.30	0.06	0.72	0.03	0.67	0.04	0.51	0.01	0.56	0.07
Hispanic/Latino	0.22	0.07	0.38	0.08	0.64	0.05	0.29	0.06	0.70	0.04	0.70	0.05	0.52	0.01	0.55	0.08
White	0.12	0.09	0.25	0.10	0.54	0.08	0.16	0.08	0.71	0.06	0.79	0.10	0.51	0.01	0.41	0.10
*p* value	0.03		0.16		0.12		0.02		0.26		0.42		0.71		0.05	
Age																
*p* value	0.16		0.41		0.45		0.62		0.51		0.33		0.67		0.35	

*SE* standard error

Bolded differences are significant *p* < .01 to account for separately evalutaing reponses in the 8 domains

^a^Each quality domain score could range from 0 to 1, with 1 being the highest possible. The higher the number, the higher report of screening. See text in methods section for details on scaling as well as “Appendix”. Each analysis controlled for the other covariates (intervention/control; gender; race/ethnicity; age)

Beyond the differences between intervention and control sites, additional analyses included a measure of practice consistency using intra-class correlation with confidence intervals (data not shown, available upon request). All 95 % confidence intervals for the ICCs contained the value 0, leading us to conclude that adolescents’ reports were largely unrelated to the clinic attended, after accounting for the modeled variables.

## Discussion

This study shows that through stakeholder participation and engagement during planning and implementation, diverse clinics can expand the use of health risk assessments (HRA) with adolescents, and in turn lead to improved adolescent recall of high quality care. Difficulties in implementing comprehensive adolescent HRA are well documented, and include inadequate time, lack of and/or perceived lack of privacy and confidentiality, and lack of knowledge how best to administer an HRA [2, 15]. While a few studies have shown that computer-based entry may decrease incidental disclosure risk and increase the veracity of sensitive question responses [23], to our knowledge this is the first study that has adapted HRAs to an online format with the addition of individualized, geo-coded community-based resources that adolescents could afford and access. Our feasibility study is unique in its incorporation of adolescent, provider, and clinic staff input into the design and implementation of the health information technology (HIT)-enhanced HRA. Further, the goal of the intervention was met as evidenced by the successful implementation of the HIT-enhanced HRA in a heterogeneous PBRN that included a variety of primary care settings and a diverse patient population.

Importantly, this study also met its secondary goal. HIT-enhanced HRAs improved essential aspects of quality of care as measured by both practice process changes and improved patient-reported outcomes. Adolescents in intervention practices reported increased rates of private and confidential care, both important aspects of adolescent care that are often perceived as difficult to operationalize during routine clinical care yet implemented well in this study [4, 20, 36]. Additionally, adolescents perceived higher rates of screening and counseling for emotional issues and relationships. It is possible that the HRA prompted providers to ask more in-depth or focused questions and offer additional counseling. Further, answering emotional and relationship questions may have encouraged adolescent self-reflection and initiation of related discussions [8]. It is important to note that the effectiveness of HRAs for reducing adolescent risk remains undecided as there is mixed evidence that assessing adolescents’ risks leads to measurable risk reduction [7, 19, 26, 34], potentially because of sporadic practice implementation [14, 26], and because, not all adolescents, especially those at highest risk, are screened due to the low rate of annual adolescent well-visits [6, 31]. Nonetheless, the recall of higher quality care amongst the adolescents is independently an important outcome and may in future studies reveal improved health outcomes and risk reduction. A next step would be a study powered to detect potential differences in all domains to evaluate the true effect of the HIT-enhanced HRA.

In addition to the processes and outcomes, quality care must include addressing the needs of the highest risk patients. While the moderate number of clinics prevented full assessment of the differences in the intervention by race/ethnicity, gender or age, we were still able to examine these characteristics on overall quality of care for participants in both study groups. In contrast to prior studies, we found no variations in reports of private, confidential care by gender or race/ethnicity except for a significantly higher level of perceived helpfulness of screening and counseling by females. In other words, all adolescents, not just those traditionally at risk for health disparities due to race, ethnicity or gender [15, 32, 35] benefitted from this approach to preventive care.

This study has several limitations that merit attention. Overall, we believe that this feasibility study is generalizable to other settings given the breadth in clinic types and diversity of enrolled patients. Yet it is possible that the success of this project was due at least in part to the efforts of the study faculty and staff who worked on this project and their relationships within the context of the PBRN, rather than the HIT-enhanced HRA and implementation strategy alone. Having study staff assigned to clinics may help practice implementation of any measure and should be tested in future studies. In addition, we learned that some patients, parents and providers were not as comfortable using the tablet-based technology, or were only interested due to the tablet itself. Additionally, the HIT-aspect of the HRA remains untested; it is unclear if the tablet format was a novelty compared to paper-based HRAs, or if the specific community resources that were linked to high-risk responses facilitated the recall of higher quality of care. Some providers also found it inconvenient to log into an additional online system. Integrating this online system with practice electronic health records might have facilitated adherence, but was impractical to implement due to the number of different electronic systems used within the PBRN. Selection bias may have resulted from our use of a convenience sample of practices for the pilot. Another limitation is the lack of a systematic assessment of the providers’ perspectives. However, anecdotally we learned that while the HIT-enhanced HRA did not save providers any time, they felt like they were providing more comprehensive care and learned about more aspects of their adolescent patients’ lives, leading to improved quality of care. Additionally, as with any study with non-random allocation to treatment, the potential for residual confounding remains. A final limitation is that the follow up survey, the YAHCS, is a patient report based on recall. However, we believe that if an adolescent does not recall a specific component of screening or counseling then the counseling likely had limited utility. Despite these limitations, we believe that the findings of feasible HIT-enhanced HRA screenings with adolescents subsequently reporting higher quality of preventive care are generalizable to other populations given that the study employed stakeholder involvement and multiple clinic sites with diverse subjects.

Quality adolescent health care requires systematic health risk assessment to ensure universal screening for high-risk behaviors across all populations of adolescents. While past experiences with such assessments have yielded low rates of adherence [2, 15], the combination of stakeholder engagement and technology-based support systems, such as screening tools similar to those used in this study, provide a structure for the implementation of systematic screening. Further, as this pilot study shows, systematic health risk assessment leads to a higher recollection of quality care, at least in the important domains of emotions and relationships and private and confidential care. Given that recommendations for health risk assessment are standard policy from the American Academy of Pediatrics, the Society for Adolescent Health and Medicine, and the American Medical Association, this feasibility study seems to reduce known barriers to screening in the primary care setting allowing a broader, systematic, standardized, and confidential HRA implementation. While it is unknown if the risks identified by this HRA screening tool and the subsequent, geo-coded counseling that the adolescents received actually changed behavior, a critical outcome was the adolescents’ recall of high quality care. Future work might focus on refining the intervention and its implementation, with particular attention to differential uptake by providers. While the tablet-based technology will never replace the important face-to-face time between provider and patient, it can promote an essential standard of care for all adolescents.

## Appendix: Technical Appendix of YAHCS Scaling

The YAHCS survey contains eight quality domains, that asks adolescents about how their provider performed screening and counseling about: (1) prevention of risky behaviors (2) sexual health; (3) weight, diet and exercise; (4) behavioral health; (5) provision of private and confidential care; (6) helpfulness of the counseling; (7) communication and experience of care; and (8) overall experience [27, 28]. For display in Table 3 and comparability, we scaled the domains to fall between 0 and 1 (with scores closest to 1 representing higher levels of screening performed) in three ways.  For domains 1–5 and 8, we calculated the arithmetic average scores of all questions within each domain because these were binary outcome variables. 

Domains 6 and 7 did not have binary outcomes per question, but rather ordinal levels. For these, we took the sum of the levels for each question answered and divided it by the total number of questions answered. From this result we subtracted 1 then divided by the number of non-missing levels each question had minus 1. That is:

$$Scaled\,Score = \frac{(\sum\,levels/n\,questions\,answered) - 1}{n\,levels\,per\,question - 1}$$

In domain 6, all questions had 4 levels so we simply calculated $${\raise0.7ex\hbox{${\left( {(\sum\,levels/n\,questions\,answered) - 1} \right)}$} \!\mathord{\left/ {\vphantom {{\left( {(\sum\,levels/n\,questions\,answered) - 1} \right)} 3}}\right.\kern-0pt} \!\lower0.7ex\hbox{$3$}} .$$

However, in domain 7 six questions had 5-level responses and one question had an 11-level response. In this case we weighted the scaled scores such that:

$$Scaled\,Score = \frac{6}{7} \times \frac{(\sum\,levels/n\,answered) - 1}{4} + \frac{1}{7} \times \frac{(\sum\,levels/n\,answered) - 1}{10}$$

All of these scaled scores resulted in a score that was between 0 and 1.

## Abbreviations

HIT: Health information technology
HRA: Health risk assessment
YAHCS: Young Adult Health Care Survey
